# Increased Expression of Toll-Like Receptors by Monocytes and Natural Killer Cells in ANCA-Associated Vasculitis

**DOI:** 10.1371/journal.pone.0024315

**Published:** 2011-09-06

**Authors:** Henko Tadema, Wayel H. Abdulahad, Coen A. Stegeman, Cees G. M. Kallenberg, Peter Heeringa

**Affiliations:** 1 Department of Rheumatology and Clinical Immunology, University of Groningen, University Medical Center Groningen, Groningen, The Netherlands; 2 Department of Nephrology, University of Groningen, University Medical Center Groningen, Groningen, The Netherlands; 3 Department of Pathology and Medical Biology, University of Groningen, University Medical Center Groningen, Groningen, The Netherlands; Centre de Recherche Public de la Santé (CRP-Santé), Luxembourg

## Abstract

**Introduction:**

Toll-like receptors (TLRs) are a family of receptors that sense pathogen associated patterns such as bacterial cell wall proteins. Bacterial infections are associated with anti-neutrophil cytoplasmic antibodies (ANCA)-associated vasculitis (AAV). Here, we assessed the expression of TLRs 2, 4, and 9 by peripheral blood leukocytes from patients with AAV, and investigated TLR mediated responses *ex vivo*.

**Methods:**

Expression of TLRs was determined in 38 AAV patients (32 remission, 6 active disease), and 20 healthy controls (HC). Membrane expression of TLRs 2, 4, and 9, and intracellular expression of TLR9 by B lymphocytes, T lymphocytes, NK cells, monocytes and granulocytes was assessed using 9-color flowcytometry. Whole blood from 13 patients and 7 HC was stimulated *ex vivo* with TLR 2, 4 and 9 ligands and production of cytokines was analyzed.

**Results:**

In patients, we observed increased proportions of TLR expressing NK cells. Furthermore, patient monocytes expressed higher levels of TLR2 compared to HC, and in a subset of patients an increased proportion of TLR4^+^ monocytes was observed. Monocytes from nasal carriers of *Staphylococcus aureus* expressed increased levels of intracellular TLR9. Membrane expression of TLRs by B lymphocytes, T lymphocytes, and granulocytes was comparable between AAV patients and HC. Patients with active disease did not show differential TLR expression compared to patients in remission. *Ex vivo* responses to TLR ligands did not differ significantly between patients and HC.

**Conclusions:**

In AAV, monocytes and NK cells display increased TLR expression. Increased TLR expression by these leukocytes, probably resulting from increased activation, could play a role in disease (re)activation.

## Introduction

Anti-neutrophil cytoplasmic antibodies (ANCA) associated vasculitides (AAV) are a group of severe autoimmune disorders, characterized by necrotizing inflammation of small- to medium-sized blood vessels. ANCA in AAV are mainly directed against proteinase 3 (PR3) or myeloperoxidase (MPO) [1], [2]. The etiology of AAV is unknown, although bacterial and viral infections have been linked to the onset and development of AAV [3], [4]. Sixty-three percent of patients with Wegener's granulomatosis (WG), a prototype AAV, are chronic nasal carriers of the bacterium *Staphylococcus aureus*, and carriage is associated with an increased risk for relapses [5], [6]. Interestingly, anti-bacterial treatment reduces the risk for relapses, and can be part of maintenance therapy [7], [8]. Taken together, these findings point towards a link between infection and autoimmunity, but the underlying mechanisms are still unknown.

Toll-like receptors (TLRs) are a family of receptors that sense pathogen associated molecular patterns (PAMPs) such as bacterial cell-wall components and DNA [9]. TLRs are expressed by a variety of cell types, including monocytes, B lymphocytes and granulocytes, as well as epithelial and endothelial cells [10]–[13]. TLR ligation results in the production of proinflammatory cytokines, increased antigen presentation, antibody production, proliferation, and differentiation [14]–[16]. Furthermore, TLR signaling has been suggested to play a role in the regulation of B-cell tolerance and the development of regulatory B-lymphocytes [17], [18]. TLRs being important during bacterial infection are the cell-surface receptors TLR2 and TLR4, sensing lipoproteins and liposaccharides, respectively [19]. Studies in knock-out mice have shown an important role for TLR4, and a crucial role for TLR2 in the defense against Gram-positive bacteria such as *Staphylococcus aureus*, by regulating proinflammatory cytokine and chemokine release, and leukocyte recruitment [20], [21]. In addition to cell wall proteins, bacterial DNA is of interest in the context of bacterial infections. Unmethylated cytosine-phosphate-guanine (CpG) motifs are prevalent in bacterial DNA, and can be recognized by TLR9, inducing proinflammatory responses [22]. Recent studies show that CpG motifs can trigger the production of autoantibodies *in vitro* [15], [23], [24].

A role for TLRs during autoimmune inflammation is likely. Increased TLR expression has been observed in synovial tissue and synovial macrophages from patients with rheumatoid arthritis [25], and involvement of TLR4 has been demonstrated in a mouse arthritis model [26]. In patients with systemic lupus erythematosus, elevated intracellular TLR9 has been found in B lymphocytes, possibly reflecting increased B lymphocyte activation in these patients [27]. Animal studies have shown both aggravating as well as protective effects of TLR ligands in a lupus model demonstrating the complexity of the system [28]. In large vessel vasculitis, it has been shown that different TLR ligands can induce distinct vasculitis types [29].

Limited data is present about TLRs in AAV. Two studies investigated whether anti-PR3 antibodies influence the expression of TLRs on human monocytes, albeit with conflicting results [30], [31]. In mice, it has been shown that lipopolysaccharide (LPS) aggravates anti-MPO antibody induced injury in a TLR4 dependent manner [32], [33], and a recent study showed that TLR2 and TLR9 ligands can drive the development of anti-MPO autoimmunity as well [34].

Given the association of AAV with infections it is highly likely that TLR mediated effector mechanisms play a role in AAV pathogenesis, but data on leukocyte TLR expression in these patients is lacking. Therefore, the current study was designed to characterize the expression of membrane TLR2, TLR4, and TLR9, and intracellular TLR9 by peripheral blood leukocytes in AAV patients, in comparison to age-matched healthy individuals. Using flowcytometry, TLR expression by B lymphocytes, T lymphocytes, NK cells, monocytes, and granulocytes was determined. TLR expression was related to serum ANCA titers and nasal carriage of *Staphylococcus aureus*. In addition, we studied whether ANCA priming influences TLR expression, and determined *ex vivo* responses to TLR ligands by peripheral blood leukocytes.

## Materials and Methods

### Patients

Heparinized blood was collected from 38 AAV patients (mean age of 60.6±13.8 years) and 20 matched healthy individuals (mean age of 53.6±13.7 years). The study was approved by the Medical Ethics Committee of the University Medical Center Groningen. Written consent was given by the participants for their blood samples to be used for research. Table 1 describes patient characteristics. Six patients had active disease at the time of inclusion. Disease diagnoses were based on the definitions of the Chapel Hill Consensus Conference [2]. Twenty-five patients received no immunosuppressive medication. Thirteen patients received maintenance therapy at the time of inclusion (prednisolone <10 mg/day, azathioprine or mycophenolate mofetil, but no cyclophosphamide). Patients were positive for circulating ANCA at the time of blood sampling.

**Table 1:** Patient characteristics.

Total number	38
PR3-ANCA/MPO-ANCA	22/16
Active/remission	6/32
Age (mean ± sd)	60.6±13.8
Diagnosis	
Wegener's granulomatosis	26
Microscopic polyangiitis	6
Necrotizing Crescentic Glomerulonephritis	6
ANCA titer (median, range)	
PR3-ANCA pts (c-ANCA)	1∶80 (1∶20–1∶640)
MPO-ANCA pts (p-ANCA)	1∶80 (1∶20–1∶320)
Nasal carriage of *S. aureus* (+/−)	13/25
Receiving / not receiving treatment	25/13

ANCA: Antineutrophil cytoplasmic antibodies.

PR3: Proteinase-3.

MPO: Myeloperoxidase.

### Flowcytometry

Membrane expression of TLR2, TLR4 and TLR9 by peripheral blood leukocytes was determined using Fluorescence-activated cell sorter (FACS). Briefly, 100 µl heparinized blood was incubated with monoclonal antibodies against membrane markers CD3 (Biolegend), CD14 (Invitrogen), CD16 (Biolegend), CD19 (BD Biosciences), CD27 (eBioscience), and CD56 (Biolegend), and toll-like receptors TLR2 (eBioscience, clone TL2.1), TLR4 (eBioscience, clone CD284), and TLR9 (Imgenex, clone IMG-305). Cells were fixed and erythrocytes were lysed using Invitrogen Fix&Perm kit. Cells were washed with PBS/BSA 1% and analyzed using a BD ™ LSR II flow cytometer. Proper isotype controls were included for TLR antibodies. The percentage of TLR expressing cells and the level of expression per cell was calculated: delta (Δ) MFI = MFI_TLR_−MFI_isotype_. Cut-off values for mTLR4 expression by HC monocytes were calculated: mean ΔMFI_TLR4_+2·SD and mean % TLR4^+^ cells+2·SD.

For intracellular TLR9 staining, cells were labeled with membrane markers, fixed and permeabilized (Invitrogen Fix&Perm kit), and incubated with anti-TLR9 antibody or an isotype control. Intracellular TLR9 expression is presented as delta-mean fluorescence intensity: ΔMFI = MFI_TLR9_−MFI_isotype_. Flowcytometry data was analyzed using Winlist 6.0 (Verity Software House).

### Effect of ANCA on TLR expression by monocytes and granulocytes

To study whether stimulation with anti-PR3 antibodies or PR3-ANCA influences TLR2 or TLR4 expression, cells from whole blood were stimulated *in vitro*, and TLR expression by monocytes and granulocytes was analyzed. Briefly, heparinized blood from healthy volunteers was diluted 1∶1 in RPMI (Cambrex Bio Science), and stimulated for 5 hours in the presence of mouse-anti-human PR3 monoclonal antibody (5 µg/ml) or isolated PR3-ANCA IgG (200 µg/ml). An irrelevant monoclonal antibody of the same isotype and healthy control IgG were used as controls. TNF-α served as a positive control. After stimulation, cells were washed, and stained with FITC-labeled monoclonal antibodies against TLR2 (eBioscience), and APC-labeled monoclonal antibodies against TLR4 (eBioscience). Cells were analyzed using FACSCalibur™ (BD). Specific monocyte and granulocyte populations were identified by forward-sideward scatter profiles, and TLR2 and TLR4 expression was analyzed on these subsets.

### Ex vivo stimulation of leukocytes by TLR ligands

Production of cytokines, and the expression of the leukocyte activation marker CD25 by peripheral blood leukocytes from AAV patients (n = 15), and HC (n = 9), were studied *ex vivo*. Whole blood (500 µl) was diluted 1∶1 in RPMI (Cambrex Bio Science), and stimulated for 24 h in the presence of 10 µg/ml Lipoteichoic Acid (Invivogen), 10 µg/ml Lipopolysachharide (Ultra pure, Invivogen), and 10 µg/ml CpG ODN 2006 (Hycult Biotechnology). After 24 h, expression of the activation marker CD25 by B-lymphocytes, monocytes, and granulocytes was analyzed using flowcytometry. Release of cytokines IL-6, IL-8, IL-10, and TNF-α into the supernatant was measured by ELISA.

### Measurement of ANCA titers

Serum ANCA titers were determined by indirect immunofluorescence on ethanol-fixed human granulocytes according to standard procedures, as previously described [35]. Titers were considered positive if equal to, or higher than 1∶20.

### Nasal carriage of Staphylococcus aureus

Patients with ANCA-associated vasculitis who visit our outpatient clinic are routinely tested for nasal carriage of *S. aureus* as described before [5]. Briefly, nasal swabs were inoculated on 5% sheep-blood and salt mannitol agar and cultured overnight. *S. aureus* was identified by coagulase and DNase positivity.

### Statistics

Statistical analyses were performed using Graphpad Prism 5.0. The non-parametric Mann-Whitney U test was used to analyze percentages of TLR^+^ NK cells, and to compare TLR expression by monocytes, in AAV patients and HC. To compare TLR expression by monocytes in S. aureus^+^ or S. aureus^−^ AAV patients, either the non-parametric Mann-Whitney U test, or the unpaired t test were used. To compare CD25 expression, and the release of cytokines between AAV patient and HC cultures, either the non-parametric Mann-Whitney U test, or the unpaired t test were used.

*P* values lower than 0.05 (2-tailed) were considered significant.

## Results

### Leukocyte Toll-like receptor expression

**Figure 1:**
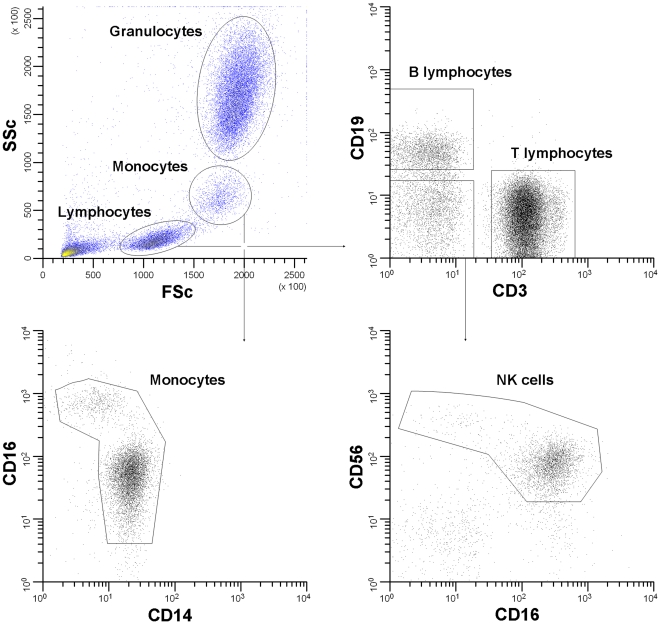
Gating strategies to distinguish between leukocyte populations. FSc-SSc profile was used to distinguish lymphocytes, monocytes and granulocytes (A). Within the lymphocyte population, B lymphocytes, T lymphocytes, and NK cells were identified using membrane markers CD19, CD3 and CD16/CD56, respectively (B, D). Monocytes were either CD14^+^/CD16^low^ or CD14^low^/CD16^+^ (C).

Leukocyte populations were distinguished according to light scatter profile and the expression of surface markers CD3, CD14, CD16, CD19, CD27, and CD56 (figure 1). All TLR expression data are summarized in table 2 and, additionally, part of the data are shown in graphs.

**Table 2:** Toll-like receptor expression by peripheral blood leukocytes.

	AAV patients (n = 38)	Healthy controls (n = 20)	*P value*
**B lymphocytes**			
mTLR2 (% pos cells)	0,51 (0–3.87)	0.84 (0–14.9)	0.26
mTLR4 (% pos. cells)	2.75 (0–13.8)	3.19 (0–11.62)	0.73
mTLR9 (% pos. cells)	2.67 (0.75–13.9)	3.82 (1.05–64.8)	0.27
iTLR9 (all B cells, ΔMFI)	10.26 (4.67–73.11)	16.07 (6.88–31.05)	**0.009**
iTLR9 (CD27^−^ B cells, ΔMFI)	11.5±4.4	13.2±4.1	0.30
iTLR9 (CD27^+^ B cells, ΔMFI)	20.0±6.8	22.1±5.6	0.37
**T lymphocytes**			
mTLR2 (% pos. cells)	0.01 (0–0.26)	0.03 (0–0.46)	0.34
mTLR4 (% pos. cells)	0.17 (0–6.6)	0.40 (0–22.7)	0.14
mTLR9 (% pos. cells)	0.23 (0–1.8)	0.36 (0–1.69)	0.33
iTLR9 (ΔMFI)	13.03 (5.6–66.1)	18.1 (11.4–47.2)	**0.006**
**NK cells**			
mTLR2 (% pos. cells)	0.48 (0–1.46)	0.20 (0–1.36)	**0.005**
mTLR4 (% pos. cells)	0.47 (0–1.84)	0.27 (0–0.77)	**0.03**
mTLR9 (% pos. cells)	0.43 (0–3.19)	0.14 (0–3.59)	**0.05**
iTLR9 (ΔMFI)	10.0 (6.1–68.7)	16.4 (6.9–35.0)	**0.008**
**Monocytes**			
mTLR2 (% pos. cells)	96.6 (58.1–99.4)	95.5 (66.0–99.3)	0.38
mTLR2 (ΔMFI)	29.7 (12.7–93.6)	19.9 (8.7–69.0)	**0.03**
mTLR4 (% pos. cells)	1.72 (0.1–65.9)	1.65 (0.1–6.88)	0.99
mTLR4 (ΔMFI)	5.82 (0–27.5)	4.19 (0–11.3)	0.10
mTLR9 (% pos. cells)	11.58 (0–77.6)	10.9 (0–51.0)	0.72
mTLR9 (ΔMFI)	6.66 (0–61.2)	5.13 (0.1–21.8)	0.87
iTLR9 (ΔMFI)	27.6 (7.8–160)	30.4 (16.0–53.4)	0.58
**Granulocytes**			
mTLR2 (% pos. cells)	5.6 (0–62.3)	6.24 (0–61.6)	0.95
mTLR2 (ΔMFI)	1.9 (0–15.3)	1.6 (0–16.4)	0.25
mTLR4 (% pos. cells)	0.31 (0–4.6)	0.36 (0–18.6)	0.87
mTLR4 (ΔMFI)	1.10 (0–9.0)	1.14 (0–13.5)	0.62
mTLR9 (% pos. cells)	0.10 (0–68.6)	0.20 (0–66.1)	0.45
mTLR9 (ΔMFI)	1.62 (0–170)	1.3 (0–28.4)	0.66
iTLR9 (ΔMFI)	12.7 (1.73–57.3)	17.1 (0–54.5)	0.22

MFI: Mean fluorescence intensity.

Membrane TLR (mTLR) expression on B- and T-lymphocytes did not differ between AAV patients and healthy controls (HC) (table 2). Intracellular expression of TLR9 (iTLR9) in the total B lymphocyte population was significantly lower in AAV patients, but did not differ between AAV patients and HC when B lymphocytes were subdivided into CD27^−^ (naïve) and CD27^+^ (memory) subsets (table 2). In T-lymphocytes, iTLR9 levels were significantly decreased in AAV patients (*P* = 0.006).

**Figure 2:**
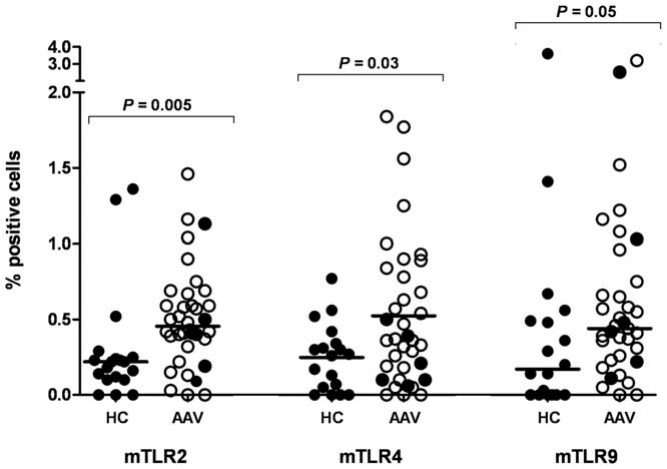
Expression of mTLR2, mTLR4, and mTLR9 by Natural killer (NK) cells. Cells in whole blood were labeled with antibodies against CD56, CD16, TLR2, TLR4, and TLR9. NK cells were gated according to light scatter profile and the expression of CD56/CD16. Proportions of mTLR2, mTLR4, and mTLR9 positive NK cells in HC and AAV patients are shown. Within patients, closed circles depict TLR expression in active patients (n = 6). Proportions of mTLR2^+^, mTLR4^+^, and mTLR9^+^ NK cells were significantly increased in AAV patients (*p* = 0.005, *p* = 0.03, and *p* = 0.05, respectively). Proportions of mTLR expressing NK cells did not differ between patients with active disease or in remission.

In both AAV patients and HC we detected low mTLR expression by NK cells. In AAV patients, proportions of mTLR2^+^, mTLR4^+^, and mTLR9^+^ expressing NK cells were increased compared to HC (*p* = 0.005, *p* = 0.03, and *p* = 0.05, respectively, figure 2). iTLR9 expression was significantly lower in AAV patient NK cells (*p* = 0.008). The percentage of NK cells – within the total lymphocyte compartment – was comparable between AAV patients and HC, and a comparable ratio between CD56^bright^ and CD56^dim^ NK cells was observed in AAV patients and HC (data not shown).

**Figure 3:**
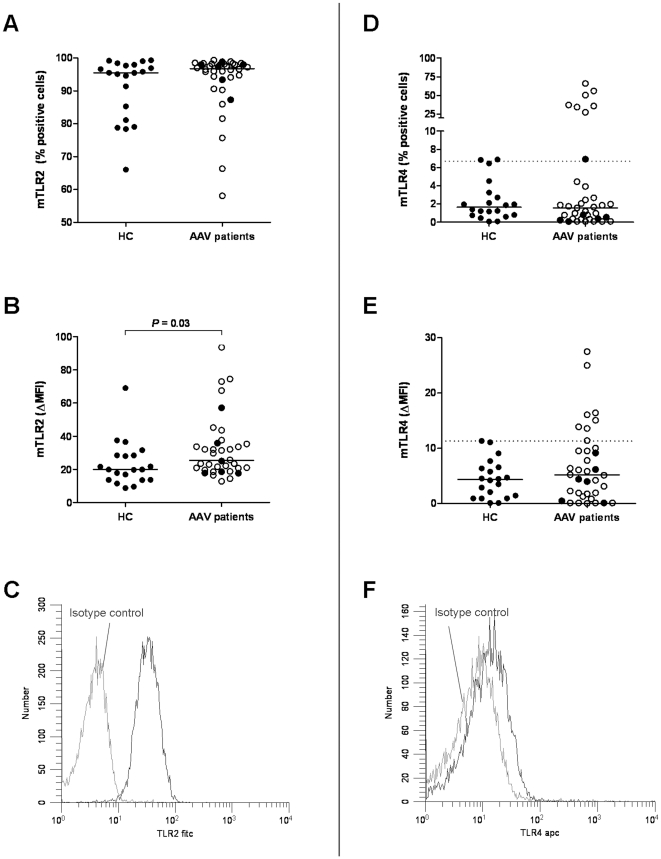
Expression of mTLR2 and mTLR4 by monocytes. Cells in whole blood were stained with antibodies against CD14, CD16, TLR2, and TLR4. Monocytes were gated according to light scatter profile and the expression of CD14/CD16. Representative histogram plots of mTLR2 and mTLR4 expression are shown in figures C and F, respectively. The proportions of mTLR2^+^ (A) and mTLR4^+^ (D) monocytes did not differ between AAV patients and HC. However, 7 AAV patients had an increased proportion of mTLR4^+^ monocytes, compared to HC (dotted line: cut-off mTLR4^+^ cells, calculated from HC). Expression levels per cell are shown in fig B (mTLR2), and E (mTLR4). mTLR2 levels were higher on AAV patient monocytes, compared to HC (*p* = 0.03), whereas mTLR4 levels did not differ significantly when comparing groups. In line with increased proportions of mTLR4^+^ monocytes, in part of the patients increased levels of mTLR4 were observed (dotted line: cut-off mTLR4 ΔMFI, calculated from HC) Within AAV patients, active patients (n = 6) are depicted with closed circles. Expression of mTLR2 and mTLR4 did not differ significantly between patients with active or quiescent disease.

mTLR2 was highly expressed by monocytes from both AAV patients and HC. Percentages of mTLR2^+^ (figure 3A) monocytes were comparable, but mTLR2 levels were increased on AAV patient monocytes (*p* = 0.03, figure 3B). Expression of both mTLR4 and mTLR9 did not differ significantly between AAV patient and HC monocytes, although 7 AAV patients had an increased proportion of mTLR4^+^ monocytes and increased level of mTLR4, compared to HC (figure 3D,E). iTLR9 levels were comparable in AAV patient and HC monocytes.

Expression of mTLRs and iTLR9 did not differ between AAV patient and HC granulocytes (table 2).

No differential TLR expression was observed in patients with active disease (n = 6), compared to patients in remission. No relation was found between TLR expression and serum ANCA titers.

### Effect of monoclonal anti-PR3 antibody and PR3-ANCA IgG priming on mTLR expression by monocytes and granulocytes

To study whether priming with anti-PR3 monoclonal antibodies or PR3-ANCA IgG influences TLR expression, we studied the expression of membrane TLR2 and TLR4 by monocytes and granulocytes after *in vitro* stimulation with monoclonal anti-PR3 antibodies, or PR3-ANCA IgG. We did not observe an increase in mTLR expression after stimulation, whereas TNF-α induced increased TLR expression (data not shown).

### Carriage of Staphylococcus aureus and TLR expression

**Figure 4:**
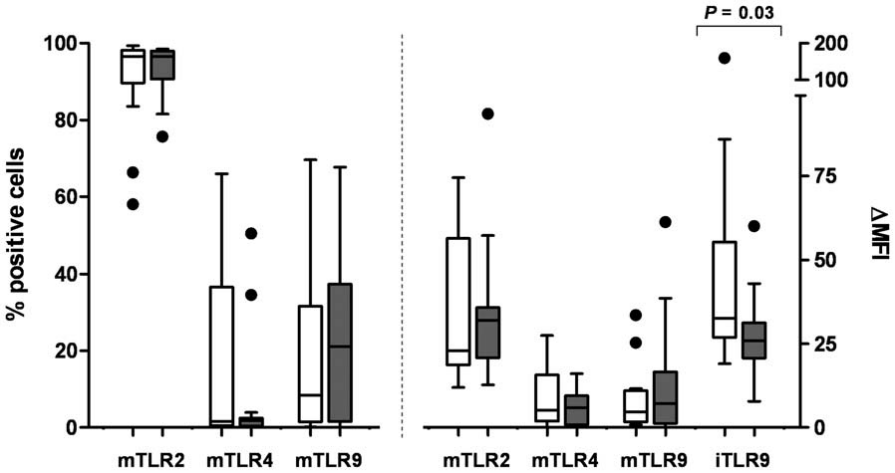
Toll-like receptor expression by monocytes from AAV patients who were nasal carriers of *S. aureus* (n = 13, white bars) and non-carriers (n = 25, gray bars). Both the proportions of mTLR^+^ monocytes (left y-axis), as well as the level of mTLR expression (right y-axis) did not differ significantly between monocytes from nasal carriers and non-carriers. Intracellular levels of TLR9 (iTLR9) were increased in monocytes from patients who were nasal carriers of *S. aureus*.

To study whether nasal carriage of *Staphylococcus aureus* influences the expression of TLRs by circulating leukocytes, the expression of TLRs was compared between AAV patients who were nasal carriers of *S. aureus* at the time of blood sampling (n = 13), and non-carriers (n = 25). Expression of TLRs by B lymphocytes, T lymphocytes, NK cells and granulocytes was comparable in nasal carriers and non-carriers (data not shown). Monocytes from nasal carriers of *S. aureus* had increased iTLR9 levels compared to non-carriers (*p* = 0.03, figure 4), and within the 7 patients with a high proportion of TLR4 expressing monocytes, 5 were nasal carriers of *S. aureus* (*p* = 0.03, chi-square).

### Whole blood stimulation with LTA, LPS, CpG

**Figure 5:**
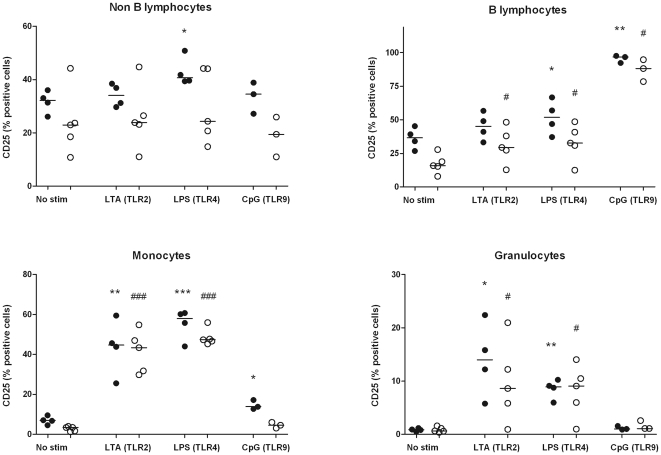
Expression of the activation marker CD25 by leukocyte subsets was analyzed after stimulation with TLR ligands. Diluted whole blood was left unstimulated or treated with Lipoteichoic acid (TLR2 ligand), Lipopolysaccharide (TLR4 ligand), or CpG-ODN (TLR9 ligand). After 24 h, expression of CD25 by different leukocyte subsets was analyzed by flowcytometry (A). Leukocyte subsets were distinguished according to light scatter profile (lymphocytes, monocytes, granulocytes) and the expression of CD19 (B lymphocytes). In both AAV patients (open circles) and HC (filled circles), increased CD25 expression was observed by monocytes and granulocytes, in response to TLR2 and TLR4 stimulation. Particularly B lymphocytes were stimulated by CpG-ODN. Significantly increased CD25 expression is depicted with * (HC) and # (AAV patients).

**Figure 6:**
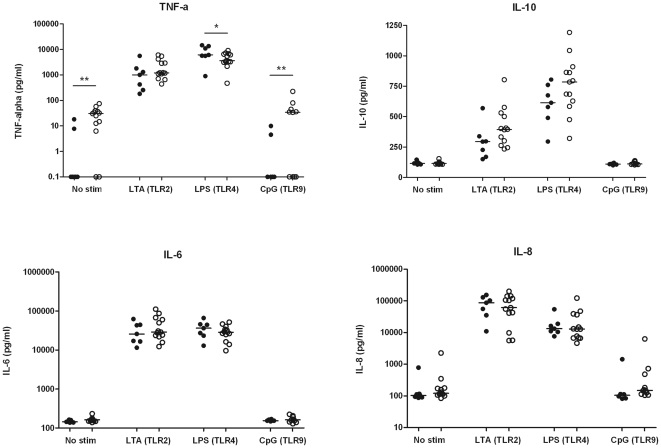
Release of cytokines after TLR stimulation in whole blood cultures was analyzed by ELISA. Diluted whole blood was left unstimulated or treated with Lipoteichoic acid (TLR2 ligand), Lipopolysaccharide (TLR4 ligand), or CpG-ODN (TLR9 ligand). Comparable total leukocyte numbers were cultured from both AAV patients and HC (data not shown). In patient cultures, spontaneous release of TNF-α was increased, but TNF-α release upon TLR4 triggering was decreased compared to HC. A trend towards increased release of IL-10 was observed in patient cultures upon TLR2 and TLR4 triggering (p = 0.09 and p = 0.06, respectively), although this did not reach statistical significance. Release of IL-6 and IL-8 was comparable in patient and HC cultures.

To study if TLR stimulation could trigger differential responses in AAV patients and HC, cells in whole blood were stimulated with specific TLR ligands. Leukocytes from patients and HC showed a comparable pattern of CD25 expression after TLR stimulation. B lymphocytes were specifically triggered by CpG, whereas monocytes and granulocytes were most responsive to TLR2 and TLR4 stimulation (figure 5). In line with these observations we observed a comparable pattern of cytokine release in response to TLR stimulation (figure 6). In the unstimulated condition, AAV patients released more TNF-α than HC, but upon LPS stimulation, AAV patients released significantly less TNF-α (*p* = 0.04). Interestingly, AAV patients tended to release more IL-10 in response to LTA, although this did not reach statistical significance (*p* = 0.06).

## Discussion

TLRs sense specific bacterial components, and bacterial infections have been associated with AAV [3], [9], [36]. The expression levels of TLRs on peripheral leukocytes and the functional consequences of TLR activation have not been studied yet in AAV. Our study is the first to characterize TLR expression by circulating B lymphocytes, T lymphocytes, NK cells, monocytes, and granulocytes from AAV patients. We observed increased expression of membrane TLR2 (mTLR2) by monocytes, and, in a subset of patients, increased mTLR4. Furthermore, patients had increased proportions of TLR-expressing NK cells. Increased TLR expression by monocytes was found in nasal carriers of *Staphylococcus aureus*. Stimulation with neither anti-PR3 monoclonal antibodies nor anti-PR3 IgG influenced TLR expression by monocytes and granulocytes, and *in vitro* responses to TLR stimulation did not differ significantly between AAV patients and HC.

We observed increased expression of mTLR2 by monocytes in patients with AAV. TLR2 senses components from the Gram-positive bacterial cell wall such as peptidoglycan and lipoteichoic acid [37]. Monocytes increase mTLR2 expression upon stimulation by proinflammatory cytokines and TLR-ligands [12], and our finding may well reflect increased activation of monocytes, as reported previously in AAV [38], [39]. It has been described that proinflammatory monocytes (CD14^dim^CD16^++^) express higher mTLR2 levels than CD14^+^CD16^dim^ monocytes [40], and, therefore, we studied whether AAV patients had higher proportions of proinflammatory monocytes. However, proportions of proinflammatory monocytes were comparable between AAV patients and HC (data not shown), indicating increased mTLR2 expression by all monocytes. mTLR4 levels on patient monocytes did not differ significantly from HC, but, interestingly, 7 AAV patients had increased proportions of mTLR4^+^ monocytes when compared to HC. This apparent discrepancy between mTLR2 and mTLR4 expression by monocytes may be explained by difference in expression kinetics of these TLRs. Indeed, previous studies have shown that mTLR4 levels decrease relatively fast after monocyte activation, whereas mTLR2 levels remain high for a longer period of time [12].

The increased proportion of TLR-expressing NK cells in AAV patients is of interest. NK cells express mRNA for TLR1–10, and produce TNF-α in response to several TLR ligands including peptidoglycan, LPS and poly I∶C [41]. The increased proportions of mTLR expressing NK cells thus may reflect a pro-inflammatory environment in AAV patients. Within the total NK cell population, two NK cell subsets can be distinguished, based on the expression level of CD56. CD56^bright^ NK cells can produce cytokines such as interferon-γ and tumor necrosis factor-α, whereas CD56^dim^ NK cells have cytotoxic capactity [42]. We analyzed whether NK cell numbers, or the CD56^bright^/CD56^dim^ ratio differed in AAV patients, but found these characteristics to be comparable with HC (data not shown). NK cells play an important role during bacterial and viral infection, and are critically required during host defense against *S. aureus* infection in the lung [43]. However, we did not observe increased proportions of TLR expressing NK-cells in nasal carriers of *S. aureus*.

Intracellular TLR9 (iTLR9) is involved in the recognition of CpG motifs from bacterial DNA [44]. We observed decreased iTLR9 in B lymphocytes, T lymphocytes, and NK cells from AAV patients, whereas iTLR9 levels in monocytes and granulocytes were comparable in patients and HC. Increased iTLR9 expression has been shown in memory B lymphocytes and, therefore, we additionally analyzed iTLR9 levels in naïve (CD27^−^) and memory (CD27^+^) B lymphocytes. In accordance with previous reports we found higher iTLR9 levels in memory B lymphocytes, compared to naïve B lymphocytes [45]. Decreased iTLR9 levels in patients are in line with lower proportions of memory B lymphocytes in AAV patients. Whether decreased iTLR9 levels in T lymphocytes and NK cells reflect differential T lymphocyte and NK cell subsets in patients as well, could not be determined with the antibody-panel that was used in this study. T lymphocytes have been studied extensively in AAV, and are shifted towards an effector memory T-cell phenotype [46] but whether these cells express lower levels of iTLR9 is not known.

As mentioned earlier, patients with Wegener's granulomatosis are often chronic nasal carriers of *S. aureus* and carriage is associated with an increased risk for relapses. TLRs are important orchestrators of the immune system during pathogen invasion [20], [21], and altered TLR expression has been reported during bacterial infections [47], [48]. Therefore, we compared TLR expression in nasal carriers of *S. aureus* and non-carriers. We observed increased iTLR9 levels in monocytes from *S. aureus* carriers, and interestingly, 5 out of 7 patients with increased proportions of monocytes expressing mTLR4 were *S. aureus* ^+^ at the moment of inclusion. These observations suggest increased activation of monocytes in nasal carriers of *S. aureus* and it will be important to follow-up on these patients and monitor the development of relapses.

Key players in AAV are the ANCA autoantibodies that are capable to activate monocytes and granulocytes [49], [50]. Uehara *et al.* found highly increased expression of TLR2, TLR3, TLR4, TLR7, and TLR9 by peripheral blood mononuclear cells after stimulation with mouse anti-human PR3 monoclonal antibody [30]. This suggests that ANCA may increase the sensitivity to TLR ligands by influencing TLR expression in AAV. However, conflicting data exist, since Hattar *et al.* found no effect of ANCA priming on monocyte TLR expression [31]. In line with these results we found no effect of priming with anti-PR3 monoclonal antibodies or isolated PR3-ANCA IgG on TLR expression by monocytes and granulocytes either. In addition, no relation between serum ANCA titers and leukocyte TLR expression was observed.

Additional to the analysis of TLR expression, we compared *ex vivo* responses to TLR ligands between AAV patients and HC. Several studies have reported increased proinflammatory responses to TLR ligands in various diseases [47], [51], [52]. However, although minor differences were observed, responsiveness to TLR ligands in AAV patients and healthy controls was largely comparable.

In this study, we observed increased expression of mTLR2, and partly mTLR4, by circulating monocytes from AAV patients. Furthermore, increased proportions of mTLR2^+^, mTLR4^+^, and mTLR9^+^ NK cells were found in AAV patients. This may reflect increased activation of these cells in AAV. No relation was found between ANCA titers and TLR expression, and *in vitro* stimulation with monoclonal anti-PR3 antibody or isolated PR3-ANCA IgG, did not alter leukocyte TLR expression either. Interestingly, we observed increased TLR expression by monocytes from nasal carriers of *S. aureus*, indicating increased activation of these cells.

These findings warrant further investigation of TLRs in relation to AAV. Local expression and function of TLRs at sites of inflammation will need to be investigated. Functional studies should point out whether TLRs play a role in the response to bacterial triggers and can contribute to disease activation and relapses in AAV.
